# Oral Midazolam Premedication for Children Undergoing General Anaesthesia for Dental Care

**DOI:** 10.1155/2009/274380

**Published:** 2009-04-13

**Authors:** Saad A. Sheta, Maha AlSarheed

**Affiliations:** ^1^Division of Anesthesiology, Department of Maxillofacial Surgery and Diagnostic Sciences, College of Dentistry, King Saud University, P.O. Box 60169, Riyadh 11545, Saudi Arabia; ^2^Division of Pediatric Dentistry, Department of Preventive Dental Sciences, College of Dentistry, King Saud University, P.O. Box 60169, Riyadh 11545, Saudi Arabia

## Abstract

*Objectives*. To assess the efficacy and safety of injectable midazolam administered orally in 3 different doses in children undergoing complete dental rehabilitation under GA. *Subjects and Methods*. 60 children aged 2–6 years were enrolled in the study. The children were randomly assigned to one of 3 groups and received orally 0.5, 0.75, or 1.0 mg/kg of injectable midazolam mixed with apple juice 30 minutes before separation from parents. The following measurements were assessed: patient's acceptance of the medication, reaction to separation from parents, sedation scores, and recovery conditions. *Results*. More children were comfortable with parent separation in the group that received the 1.0 mg/kg dose (90%) compared to the group that received the 0.75 mg/kg dose (75%) and the group that received the 0.5 mg/kg dose (55%). The number of children who had desirable sedation was similar in the 0.75 mg/kg and 1.0 mg/kg dose groups. Twenty five percent of the children in the group that received the 0.5 mg/kg dose did not allow venepuncture before induction of GA, and induction of GA was poor for 20% of the children in this group. An increasing number of children scored excellent in terms of ease of venepuncture in 0.75 mg/kg dose group (10%) and in the 1.0 mg/kg dose group (20%) and in terms of induction of GA, 25% and 35%, respectively. Recovery of spontaneous ventilation and extubation was delayed by over 15 minutes in 2 children in the 1.0 mg/kg dose group. *Conclusion*. The dose of 0.75 mg/kg of injectable midazolam given orally as premedication is acceptable, effective, and safe.

## 1. Introduction

It is generally agreed that most children who are undergoing medical procedures and who are fearful and uncooperative can and should be managed with behavioral (nonpharmacologic) management techniques. Unfortunately, a small percentage of pediatric patients cannot be successfully managed solely with these techniques [1]. When behavioral management strategies fail, some form of pharmacologic sedation or anesthesia becomes a valuable and necessary alternative. Children who are undergoing dental procedures and who are extremely uncooperative, fearful, anxious, or physically resistant with no expectation of behavior improvement are candidates for general anaesthesia (GA) [2].

An effective premedication may facilitate a smoother induction of GA with minimal hemodynamic alterations and minimize the emotional trauma in children undergoing surgery. Premedication is best given by an oral route in children as children often exhibit an exaggerated psychological response to a needle, and it is easier to distribute a medication orally than to use nasal or rectal routes [3, 4].

Midazolam, with its rapid onset and relatively short duration of action, has proven to be a useful premedication to decrease preoperative anxiety and facilitate separation from parents with fewer unwanted side effects [5]. However, an oral preparation of midazolam is not commercially available in some countries. Doses of injectable midazolam mixed with apple juice were used in this study as an oral premedication. 

The objectives of this study were to assess the efficacy and safety of oral injectable midazolam in three different doses and to determine the optimal dose as a premedication in children undergoing complete dental rehabilitation.

## 2. Subjects and Methods

The study protocol received institutional approval from the Ethics Committee of the College of Dentistry Research Center (CDRC) of the King Saud University in Riyadh, Saudi Arabia. Written informed consent was obtained from at least one of the parents, and verbal consent from the child was sought whenever possible. 

Sixty children were selected from those visiting Pediatric Dentistry Clinics in the College of Dentistry, King Saud University. Healthy children (ASA I & II) aged 2 to 6 years within normal range of weight who were scheduled for complete dental rehabilitation under general anesthesia (GA) on an outpatient basis were enrolled in the study protocol. Children who were referred to GA, according to the protocol adopted in Dental College, had a rating of 1 (definitely negative) according to Frankl behavior category scales rating [6]. These children refused treatment, were extremely uncooperative, were anxious, cried forcefully or fearfully, or displayed any overt evidence of extreme negativism.

Children were excluded from the study if they were hemodynamically or respiratory unstable, had any mental retardation or physical disabilities, were under treatment with sedatives or anticonvulsants, or if their parents refused to allow them to participate.

The study was designed as randomized double blinded study. The original study plan was to include a control group (placebo group). However, in a pilot study, the first few children who received no premedication were anxious and agitated. We believed that it was not ethical to expose them to such suffering and decided to exclude the control group from the study. Therefore, children were randomly assigned by sealed envelope technique to one of three groups, each containing 20 participants. 

Groups A, B, and C received an oral administration of 0.5 mg/kg, 0.75 mg/kg, and 1.0 mg/kg, respectively, of injectable midazolam mixed in apple juice, 30 minutes before separation from parents (premedication time). Oral midazolam is not available commercially in Saudi Arabia. An injectable 5 mg/mL preparation of midazolam was used, thus limiting the total volume mixed with a double volume of apple juice.

The drug was prepared and administered by the pre-operative room nurse based on the patient's assigned group, so that the anesthesiologists, dentists, and the Post Anesthesia Care Unit (PACU) nurse remained blinded to the groups. The PACU nurse was responsible for recording participants' heart rate, blood pressure, respiratory rate, oxygen saturation (SaO_2_), reaction to separation from parents, and sedation scores including ease of venepuncture. The PACU nurse also observed induction of GA. 

All children received a standardized GA by the same anesthesiologist. The anesthesiologist used sevoflurane or propofol induction (if an intravenous line could be established before induction) in a dose of 2 mg/kg. Thereafter, 2 *μ*g/kg of fentanyl and 0.5 mg/kg of atracurium were injected to facilitate tracheal intubation. After nasal preparation with a vasoconstrictor (oxymetazoline), a nasal endotracheal tube of the appropriate size was inserted. Then, a pharyngeal throat pack was introduced. Sevoflurane was the inhalational anesthetic used for maintenance of anesthesia. Ventilation was controlled to maintain normocapnia (PetCO_2_ 32–38 mm Hg). Patients' electrocardiogram, noninvasive arterial blood pressure, pulse oximetry, temperature, and inspiratory and expiratory gas concentrations were monitored as part of standard GA procedure. 

For postoperative analgesia, an injection of 0.5 mg/kg of ketorolac was given intramuscularly 30 minutes before the anticipated end of surgery and repeated after every 6–8 hours. Local anesthetic infiltration (xylocaine 20 mg/mL and adrenaline 12.5 *μ*g/mL) was administered by the dentist before tooth extraction. The dentist's confirmation that bleeding at the sites of extraction was controlled defined the end of surgery. The throat pack was then removed, and the inhalational anesthetics were discontinued. Reversal of muscle relaxation and recovery of neuromuscular function were confirmed using the train-of-four (TOF) method [7]. A nerve stimulator applied to the ulnar nerve was used for intraoperative monitoring of muscle relaxant. 

The fresh gas flow was increased to 10 L/min in all 3 groups. Tracheal extubation was performed when normoventilation was achieved and the patients regained gag or cough reflex. Thereafter, all patients were transferred to the PACU. The duration of exposure to volatile anesthetics was recorded by an observer blinded to anesthetic management.

Recovery monitoring was performed for all patients during their stay in the PACU according to the standard protocol of our institution. No patient was returned to the ambulatory unit earlier than 30 minutes after completion of the procedure.

GA induction, recovery conditions, and side effects were recorded by an independent PACU nurse who was also blinded to the premedication dose.

### 2.1. Assessments

#### 2.1.1. Patient's Acceptance of the Medication

Acceptance of the medication was defined as swallowing without immediate regurgitation. The preoperative room nurse was asked to add extra patients to the same group if any of the patients did not accept the medication or vomited soon after ingestion. Thus, the number of patients in each group remained equal for further observation.

#### 2.1.2. Reaction to Separation from Parents

The response of the children when taken away from the parents was recorded as the end point of premedication. It was graded as inconsolable cry, complaining, quiet but awake, or sleepy.

#### 2.1.3. Sedation Scores

The degree of sedation, when the child was first seen in the operating room (OR) and at the end after reversal of residual paralysis, was based upon the 5 points sedation score, given in Table 1 [8].

#### 2.1.4. Ease of Venepuncture and Induction of GA

Both ease of venepuncture and induction of GA were graded as poor, fair, good, or excellent depending upon the individual child's response in the OR.

#### 2.1.5. Recovery Conditions

We looked for spontaneous ventilation after giving the reversal and noted the time required for establishing adequate spontaneous ventilation followed by endotracheal extubation. We noted the need to supplement oxygen for maintaining >95% SaO_2_.

#### 2.1.6. Statistical Analysis

Statistical analysis was performed using SPSS version 13 (SPSS Inc., Chicago, Fl, USA). Demographic variables and duration of anesthesia were compared using ANOVAs test, whereas the results were compared within groups using Chi Square analysis. Also, a nonparametric test was used to compare continuous data values. A *P*-value of <.05 was considered statistically significant.

## 3. Results

All children in the pilot study (without premedication) were extremely anxious and cried inconsolably when they were separated from their parent(s). When these children were first seen in the OR, venepuncture was impossible, and we decided to induce GA and to exclude them from the study (Table 2).

All treatment groups were comparable with respect to ASA status, gender, age, weight, and duration of anesthesia as shown in Table 3. During the premedication time, none of the patients in the study had an incidence of bradycardia (heart rate <20% baseline), hypotension (mean blood pressure <20% of baseline) or desaturation episodes (O_2_ saturation <95%) according to the preoperative room nurse.

### 3.1. Acceptance and Vomiting soon after Ingestion of the Premedication

Data showed that the acceptability of the medication was similar across the groups. One hundred percent of the children in the groups receiving 0.5 mg/kg and 1.0 mg/kg doses of midalozam and 95% in the group receiving the 0.75 mg/kg dose accepted the treatment. None of the patients in any of the groups vomited soon after swallowing the premedication.

### 3.2. Reaction to Separation from Parents

The children's reaction to being separated from their parent(s) 30 minutes after receiving premedication is displayed in Table 4. We found that none of the children in the 1.0 mg/kg dose group cried compared with 4 children (20%) in the 0.5 mg/kg dose group and one child (5%) in the 0.75 mg/kg dose group. The percentage of children who appeared uncomfortable (study nurse recorded that they were crying or complaining) were the highest in the 0.5 mg/kg dose group (45%). Only 25% of the children in the 0.75 mg/kg dose group and 10% of the children in the 1.0 mg/kg dose group appeared uncomfortable. Thus more children were comfortable (study nurse recorded they were sleepy or quite but awake) in the 1.0 mg/kg dose group (90%) compared with the 0.75 mg/kg dose group (75%) and the 0.5 mg/kg dose group (55%). This difference was statistically significant between the group that received the 0.5 mg/kg dose and the group that received the 1.0 mg/kg dose (*P* < .05).

### 3.3. Level of Sedation after Premedication

All children were observed in the operating room before the induction of GA. None of the children in the 1.0 mg/kg dose group appeared to be anxious while 7 (35%) of the children in 0.5 mg/kg dose group and 3 (15%) of the children in the 0.75 mg/kg dose group appeared to be anxious (Table 5). A statistically significant difference (*P* < .005) in the percentage of patients who were satisfactorily sedated but could be aroused existed between the 1.0 mg/kg dose group (40%) and the 0.5 mg/kg dose group (15%). The number of patients who had desirable sedation (oriented or calm or drowsy but responding to verbal commands) was similar (80%) in groups receiving the 1.0 mg/kg and 0.75 mg/kg doses. Four children (20%) in the group that received the 1.0 mg/kg dose experienced deep sedation, characterized as not responding to verbal commands but to painful stimuli, whereas only one child (5%) had deep sedation in the 0.75 mg/kg dose group. There were no incidences in this study of children not responding to painful stimuli (Table 5).

### 3.4. Ease of Venepuncture and Induction of GA

Twenty-five percent of the children in the 0.5 mg/kg dose group received a rating of poor for ease of venepuncture (Table 6). None of the patients in this group received a rating of excellent on this measure. In comparison, 10% of the children in the 0.75 mg/kg dose group and 20% of the children in the 1.0 mg/kg received a rating of excellent. Four of the children in the 0.5 mg/kg dose group received a GA induction score of poor. In comparison, only 1 child in the 0.75 mg/kg dose group and none of the children in the 1.0 mg/kg dose group received a GA induction score of poor. GA induction was reported as good or excellent for 85% of children in the 1.0 mg/kg dose group, as compared to 75% of the children in the 0.75 mg/kg dose group and 45% of the children in the 0.5 mg/kg dose group. These differences were statistically significant between the 0.5 mg/kg dose group and the 1.0 mg/kg dose group as well as between the 0.5 mg/kg dose group and the 0.75 mg/kg dose group (*P* < .05).

More of the children in the 0.5 mg/kg dose group (25%) were anxious on reversal of residual paralysis than in the 0.75 mg/kg dose group and the 1.0 mg/kg dose group (5%, 0%, resp.) (Table 7). The number of children who were drowsy but arousable was the highest in the 1.0 mg/kg dose group (50%) followed by the 0.75 mg/kg dose group (20%) and the 0.5 mg/kg dose group (10%). The differences observed between the 1.0 mg/kg dose group and the 0.5 mg/kg dose group were statistically significant. Also, the percentage of children who were calm were significantly higher in the 0.75 mg/kg dose group (75%) compared to the 0.5 mg/kg dose group (25%).

### 3.5. Recovery Conditions

Most of the children in the three groups recovered spontaneous ventilation and could be extubated within 5 minutes (Table 8). However, 2 children in each of the 3 groups were extubated within 5–10 minutes of reversal. Recovery of spontaneous ventilation and extubation was delayed by over 15 minutes in 2 children in the 1.0 mg/kg dose group. Midazolam dose did not impact the overall recovery times for children in any of the 3 groups, as the average time interval from premedication to full recovery was similar for all 3 groups.

## 4. Discussion

The population of children in this study has special characteristics; they are extremely uncooperative, fearful, anxious, and physically resistant. Uncooperative children, whether due to repeated anesthesia, high anxiety, or psychological, developmental, or mental disorders, should be appropriately treated to avoid preoperative behavior problems [9]. In the nineteenth century, it was shown that up to 25% of children required physical restraint to facilitate anesthetic induction [10].

Midazolam is the most commonly used drug for premedication and is used in greater than 90% of surgical cases involving premedication in the United States [11]. The combination of the sedative and anxiolytic characteristics is believed to create a calming effect which makes children less anxious when they are separated from their parents and during mask placement [12]. Finley et al. [12] showed that a midazolam-induced decrease in anxiety was more pronounced for children with higher baseline levels of anxiety. 

Oral midazolam was found to be superior when compared with other commonly used premedications. Oral midazolam was reported to give a more predictable and effective sedation than oral diazepam [13]. It was also associated with a faster and smoother recovery, when compared with oral ketamine [14]. Patel and Meakin [15] also reported greater anxiolysis after oral midazolam (0.5 mg/kg) than after a combination of diazepam (0.25 mg/kg) with droperidol (0.25 mg/kg) or trimeprazine (2 mg/kg).

Unfortunately, commercially prepared oral formulation of midazolam is not available in many countries. A wide variety of additives have been used, with the specific choice being a matter of local practice and preference. This variation has resulted in formulas that differ from practice to practice in terms of chemical composition, active drug concentration, and pH [16]. Brosius and Bannister [16] reported that IV midazolam yields more reliable sedation and correspondingly higher plasma levels than an equivalent dose of the commercially formulated and marketed preparation. In another study, Coté et al. [17] used 3 different doses of commercial syrup in children (0.25, 0.5, and 1.0 mg/kg, up to a maximum of 20 mg) and found that the smallest dose (0.25 mg/kg) was equally as effective as the higher doses. 

The problem with injectable midazolam is that it is very bitter. In this study we used apple syrup as a carrier. We found that it is easily available and convenient to use. Mishra et al. [18] mixed IV midazolam with honey (5 times the drug volume), which was well accepted by most of their subjects. However, since this mixture is not transparent and not a liquid, there is a question if its use violates the fasting protocol for children [18]. 

Preoperative oral midazolam has proved effective in treating preoperative anxiety. Orally administered midazolam can be given in a dose of 0.25 to 1.0 mg/kg up to a total dose of 20 mg depending on the duration of surgery and the anxiety level of the child. In this study, injectable midazolam given orally as premedication was acceptable, effective, and safe in 0.75 mg/kg dose, while the 0.50 mg/kg dose was less effective. A dose of 1.0 mg/kg may produce more sedation over a 0.75 mg/kg dose but does delay recovery and may compromise safety.

Feld et al. [19] also reported a superior anxiolysis 30 minutes after a 0.75 mg/kg dose of oral midazolam as compared to 0.25 mg/kg and 0.5 mg/kg doses or placebo. Similarly, it was reported that the use of a 0.7 mg/kg dose of oral midazolam did not result in clinically respiratory depression or upper airway obstruction, but in some children caused an increased level of sedation beyond simple conscious sedation [20]. In contrast, other studies found the dose of 0.5 mg/kg to be the most effective. McMillan et al. reported no added advantage but more side effects for both 0.75 mg/kg and 1.0 mg/kg doses compared to the 0.5 mg/kg dose [21]. We found that the 0.75 mg/kg dose gave superior effects and lesser side effects than 0.5 mg/kg dose. Also, we found that increasing the dose to 1.0 mg/kg did not give any added advantage but exposed the children to more side effects, a finding corroborated by many studies [20, 21].

The conflicting results of the studies about ideal doses for midazolam as a premedicant are to be expected due to the large range of heterogeneity in the studies in regard to drug preparation, sedation scales used, patient characteristics, and time between premedication and separation from parents.

As important as the dose of premedicant is, the age of the child is also an important variable. Separation anxiety usually peaks at approximately 1 year of age, but toddlers and preschoolers may also experience the distress of separations. The extent of an individual child's risk for stress reflects genetics, personality, parenting, and previous life experience. Children 1 to 5 years of age are at the highest risk for extreme preoperative anxiety [22]. 

Clinical sedative effects are seen within 5 to 10 minutes of oral midazolam administration; the peak effect is achieved in 20 to 30 minutes [23]. Sedative effects dissipate within 45 minutes in most cases. In many studies, midazolam has been used orally at doses of 0.2–1.0 mg/kg with onset of action in 20 to 30 minutes [24]. In this study, the separation time was fixed at 30 minutes. We found a satisfactory anxiolysis in 75% of the children after a 0.5 mg/kg dose and in 90% of the children after 1.0 mg/kg dose of midazolam. On arrival in the OR, the percentage of patients who had desirable sedation (oriented, calm, or drowsy but responding to verbal command) were similar and marginally increased to 80% in the 0.5 and 0.75 mg/kg dose groups; however, four children (20%) went into undesirable deep sedation in the group that received higher dose of 1.0 mg/kg. Similar results were seen even when separation time was set to 45 minutes. 

It is believed that anxiety may be reduced by increasing the separation/premedication time. Timing should be appropriate to achieve onset time of premedicant. However, a study to determine the minimum time interval between oral midazolam (0.5–1.0 mg/kg) premedication and separation from parents to ensure a smooth separation, researchers found that children could be easily separated from their parents after only 10 minutes [23]. 

Cox et al. [25] reviewed 30 papers regarding the use of oral midazolam for premedication and concluded that it is effective in reducing both separation and induction anxiety in children, with minimal effect on recovery times. We did not observe any significant delay in recovery time after 0.5 and 0.75 mg/kg doses in children ranging from 2 to 6 years of age. Our finding of a premedication to recovery period of 3 to 4 hours is in agreement with the report of Reeves et al. [26], who reported that the mental function returns to normal after 4 hours of giving oral midazolam as a premedication. 

Anterograde amnesia is a lack of recall of events occurring from the time of administration of a drug onwards. Midazolam affects memory processes by impairing the ability to acquire new information [27]. The amnesic effect of midazolam appears to be independent of the sedation quality. Midazolam produces anterograde amnesia and may indirectly enhance the retention of material learned before treatment as a consequence of the reduced learning of information presented after the drug takes effect. The amnesic effects of midazolam generally persist for 20 to 30 minutes [28]. Midazolam was found to provide partial or complete amnesia in 90% of children undergoing bone marrow aspiration [29]. In our study the premedication time and the duration of surgery over last to time convenient to test the amnesic effect of midazolam.

We concluded that a dose of 0.75 mg/kg of injectable midazolam given orally as premedication for children undergoing complete dental rehabilitation is acceptable, effective, and safe. A dose of 0.50 mg/kg is less effective and a dose of 1.0 mg/kg may produce undue over sedation. 

## Figures and Tables

**Table 1:** Sedation Grades.

Grade	Description
I	Anxious, agitated
II	Oriented, calm, cooperative
III	Drowsy, responding to verbal commands
IV	Not responding to verbal commands but to painful stimuli
V	Not responding to painful stimuli

**Table 2:** Pilot sample results.

*Criteria*	*Results (no. of patients)*
No. of patients	5
Types of premedication	None
Reaction to separation from parents	Inconsolable cry (5)
Sedation score at OR	Extremely anxious (5)
Ease of *venepuncture*	Poor (5)
Induction score	Poor (5)

**Table 3:** Patient characteristics.

Groups	A	B	C
*Midazolam Dose*	(0.5 mg/kg)	(0.75 mg/kg)	(1.0 mg/kg)
Age mean (±SD)	4(1.026)	3.85(1.22)	4.30(1.30)
ASA I/II*	18/2	18/2	19/1
Gender (M/F)	9/11	10/10	8/12
Weight mean (kg) (±SD)	20.40(3.26)	19.85(3.52)	20.20(3.51)
Duration (min) of general anesthesia	122.75	120.75	123.50

*Number of patients

**Table 4:** Reaction to parent's separation.

Groups	A	B	C
*Midazolam Dose*	0.5 mg/kg no. (%)	0.75 mg/kg no. (%)	1.0 mg/kg no. (%)
Inconsolable cry	4(20)	1(5)	0
Complaining	5(25)	4(20)	2(10)
Total number of uncomfortable children	9(45)	5(25)	2(10)
Quiet-but-awake	10(50)	13(65)	13(65)
Sleepy	1(5)	2(10)	5(25)
Total number of comfortable children	11(55)	15(75)	18(90)^a^

^a^ *P* ≤ .05 versus group A

**Table 5:** Sedation score in OR (preoperative). RVC, responding to verbal commands.

Group	A	B	C
*Midazolam Dose*	0.5 mg/kg no. (%)	0.75 mg/kg no. (%)	1.0 mg/kg no. (%)
Anxious	7(35)	3(15)	0
Oriented, calm	10(50)	11(55)	8(40)
Drowsy-RVC	3(15)	5(25)	8(40)^a^
Not RVC but to painful stimuli	0	1(5)	4(20)
Not responding to painful stimuli	0	0	0

^a^ *P* ≤ .005* versus group A*

**Table 6:** The ease of venepuncture and induction of GA (IV/Mask).

	Ease of venepuncture			Induction score		
	A	B	C	A	B	C
	0.5 mg/kg no. (%)	0.75 mg/kg no. (%)	1.0 mg/kg no. (%)	0.5 mg/kg no. (%)	0.75 mg/kg no. (%)	1.0 mg/kg no. (%)
Poor	5(25)	3(15)	1(5)	4(20)	1(5)	0
Fair	8(40)	6(30)	4(20)	7(35)	4(20)	3(15)
Total	13(65)	9(45)	5(25)	11(55)	5(25)	3(15)
Good	7(35)	9(45)	11(55)	8(40)	10(50)	10(50)
Excellent	0	2(10)	4(20)	1(5)	5(25)	7(35)
Total	7(35)	11(55)	15(75)	9(45)	15(75)^a^	17(85)^a^

^a^ *P* ≤ .05* versus group A*

**Table 7:** Sedation score on reversal of residual paralysis. RVC, responding to verbal commands.

Groups	A	B	C
*Midazolam Dose*	0.5 mg/kg no. (%)	0.75 mg/kg no. (%)	1.0 mg/kg no. (%)
Anxious	5(25)	1(5)	0
Awake-calm	13(65)	15(75)	5(25)^a^
Drowsy-RVC	2 (10)	4 (20)	10(50)^b^
Not RVC but to painful stimuli	0	0	5(25)
Not responding to painful stimuli	0	0	0

^a^ *P* ≤ .005* versus group B*

^b^ *P* ≤ .005* versus group A*

**Table 8:** Recovery profile.

After reversal of residual paralysis		A	B	C
		0.5 mg/kg no. (%)	0.75 mg/kgno. (%)	1.0 mg/kgno. (%)
Time to spontaneous ventilation and extubation	<5	18(90)	18(90)	16(80)
(minutes)	5–10	2(10)	2(10)	2(10)
	15–60	0	0	2(10)
Time from premedication to full recovery	Hours	3.20 ± 0.41	3.17 ± 0.38	3.39 ± 0.38

## References

[1] Creedon RL, Dock M, McDonald RE, Avery DR (2004). Pharmacological management of patient behavior. *Dentistry for the Children and Adolescent*.

[2] American Academy of Pediatric Dentistry (2006). Guidelines for the elective use of conscious sedation, deep sedation and general anaesthesia in pediatric dental patients. *Pediatric Dental Journal*.

[3] Connors K, Terndrup TE (1994). Nasal vs oral midazolam for sedation. *Annals of Emergency Medicine*.

[4] Haas DA (1999). Oral and inhalation conscious sedation. *Dental clinics of North America*.

[5] Dundee JW, Halliday NJ, Harper KW, Brogden RN (1984). Midazolam. A review of its pharmacological properties and therapeutic use. *Drugs*.

[6] Frankl SN, Shiere FR, Fogels HR (1962). Should the parents remain with the child in the dental operatory?. *Journal of Dentistry for Children*.

[7] Kirov K, Motamed C, Ndoko S-K, Dhonneur G (2007). TOF count at corrugator supercilii reflects abdominal muscles relaxation better than at adductor pollicis. *British Journal of Anaesthesia*.

[8] Malviya S, Voepel-Lewis T, Tait AR, Merkel S, Tremper K, Naughton N (2002). Depth of sedation in children undergoing computed tomography: validity and reliability of the University of Michigan Sedation Scale (UMSS). *British Journal of Anaesthesia*.

[9] Watson AT, Visram A (2003). Children's preoperative anxiety and postoperative behaviour. *Paediatric Anaesthesia*.

[10] Lumley MA, Melamed BG, Abeles LA (1993). Predicting children's presurgical anxiety and subsequent behavior changes. *Journal of Pediatric Psychology*.

[11] Kain ZN, Caldwell-Andrews AA, Krivutza DM, Weinberg ME, Wang S-M, Gaal D (2004). Trends in the practice of parental presence during induction of anesthesia and the use of preoperative sedative premedication in the United States, 1995–2002: results of a follow-up national survey. *Anesthesia and Analgesia*.

[12] Finley GA, Stewart SH, Buffett-Jerrott S, Wright KD, Millington D (2006). High levels of impulsivity may contraindicate midazolam premedication in children. *Canadian Journal of Anesthesia*.

[13] Pywell CA, Hung Y-J, Nagelhout J (1995). Oral midazolam versus meperidine, atropine, and diazepam: a comparison of premedicants in pediatric outpatients. *Journal of the American Association of Nurse Anesthetists*.

[14] Alderson PJ, Lerman J (1994). Oral premedication for paediatric ambulatory anaesthesia: a comparison of midazolam and ketamine. *Canadian Journal of Anaesthesia*.

[15] Patel D, Meakin G (1997). Oral midazolam compared with diazepam-droperidol and trimeprazine as premedicants in children. *Paediatric Anaesthesia*.

[16] Brosius KK, Bannister CF (2003). Midazolam premedication in children: a comparison of two oral dosage formulations on sedation score and plasma midazolam levels. *Anesthesia and Analgesia*.

[17] Coté CJ, Cohen IT, Suresh S (2002). A comparison of three doses of a commercially prepared oral midazolam syrup in children. *Anesthesia and Analgesia*.

[18] Mishra LD, Sinha GK, Bhaskar Rao P, Sharma V, Satya K, Gairola R (2005). Injectable midazolam as oral premedicant in pediatric neurosurgery. *Journal of Neurosurgical Anesthesiology*.

[19] Feld LH, Negus JB, White PF (1990). Oral midazolam preanesthetic medication in pediatric outpatients. *Anesthesiology*.

[20] Litman RS, Kottra JA, Berkowitz RJ, Ward DS (1997). Breathing patterns and levels of consciousness in children during administration of nitrous oxide after oral midazolam premedication. *Journal of Oral and Maxillofacial Surgery*.

[21] McMillan CO, Spahr-Schopfer IA, Sikich N, Hartley E, Lerman J (1992). Premedication of children with oral midazolam. *Canadian Journal of Anaesthesia*.

[22] McCann ME, Kain ZN (2001). The management of preoperative anxiety in children: an update. *Anesthesia and Analgesia*.

[23] Levine MF, Spahr-Schopfer IA, Hartley E, Lerman J, MacPherson B (1993). Oral midazolam premedication in children: the minimum time interval for separation from parents. *Canadian Journal of Anaesthesia*.

[24] Kupietzky A, Houpt MI (1993). Midazolam: a review of its use for conscious sedation of children. *Pediatric dentistry*.

[25] Cox RG, Nemish U, Ewen A, Crowe M-J (2006). Evidence-based clinical update: does premedication with oral midazolam lead to improved behavioural outcomes in children. *Canadian Journal of Anesthesia*.

[26] Reves JG, Fragen RJ, Vinik HR, Greenblatt DJ (1985). Midazolam: pharmacology and uses. *Anesthesiology*.

[27] Veselis RA, Reinsel RA, Alagesan R, Heino R, Bedford RF (1990). Cognitive mechanism of amnesia produced by midazolam. *Anesthesiology*.

[28] Magni VC, Frost RA, Leung JWC, Cotton PB (1983). A randomized comparison of midazolam and diazepam for sedation in upper gastrointestinal endoscopy. *British Journal of Anaesthesia*.

[29] Sievers TD, Yee JD, Foley ME, Blanding PJ, Berde CB (1991). Midazolam for conscious sedation during pediatric oncology procedures: safety and recovery parameters. *Pediatrics*.

